# A Data-Driven Framework for Predicting PHBV Biodegradation-Induced Weight Loss Based on Laboratory and Real-Environment Condition Tests

**DOI:** 10.3390/polym18070897

**Published:** 2026-04-07

**Authors:** Marianna I. Kotzabasaki, Leonidas Mindrinos, Nikolaos P. Sotiropoulos, Konstantina V. Filippou, Chrysanthos Maraveas

**Affiliations:** Department of Natural Resources Development and Agricultural Engineering, Agricultural University of Athens, Iera Odos 75, 11855 Athens, Greece

**Keywords:** poly(3-Hydroxybutyrate-co-3-Hydroxyvalerate) (PHBV), polyhydroxyalkanoates (PHAs), biodegradation, weight loss, quantitative structure–activity relationship (QSAR) models, artificial intelligence, machine learning

## Abstract

Polyhydroxyalkanoates (PHAs) emerge as promising biodegradable polymers for sustainable applications, yet predicting their biodegradation behavior under different environmental conditions remains challenging. In this study, we propose a novel data-driven computational framework for predicting biodegradation-induced weight/mass loss in PHA-based materials. A comprehensive database of poly(3-hydroxybutyrate-co-3-hydroxyvalerate) (PHBV)-based formulations was manually curated by systematically collecting and harmonizing material descriptors, environmental parameters, and experimental biodegradation outcomes from laboratory- and large-scale studies conducted in soil, marine, freshwater, and compost environments. Multiple regression-based quantitative structure–activity relationship (QSAR) models were developed and rigorously validated, demonstrating high predictive performance and strong correlations between polymer structure, environmental conditions and degradation behavior. “Exposure time”, “degradation environment” and “hydroxybutyrate (HB) ratio” were identified as the most important features for weight loss. Finally, the predictive model was integrated into the Jaqpot computational platform, enabling open access and facilitating data-driven assessment and design of biodegradable polymer systems.

## 1. Introduction

The production of petroleum-derived plastics has grown exponentially over the last few decades. This production increased from 1.5 million tons in 1950 to 359 million tons in 2018, and correspondingly, the amount of plastic waste increased. Currently, households account for over 60% of the plastic waste from consumer sources. This mainly comprises single-use food packaging materials made from petroleum-derived plastics. Additionally, the consumption of plastic materials in both households and industries has exceeded the global production of plastics by up to 400 Mt/year [1]. As such, the manufacture of plastics from petroleum to meet current consumption demands poses several environmental concerns.

To mitigate environmental pollution in plastics production, polyhydroxyalkanoates (PHAs), a biobased, biodegradable material class, have emerged. Among short-chain length PHAs (scl-PHAs), the poly(3-hydroxybutyrate-co-3-hydroxyvalerate) (PHBV) has received considerable research interest due to its production by microbial fermentation processes [2], its bio-based nature, and its excellent combination of barrier properties and biodegradability, making it suitable for packaging and related applications. However, the use of PHBV as a material for real-world applications is restricted by diverse factors: the properties of the material and its performance; the varying rates of biodegradation of the material in different environments; geometries/thicknesses, and formulations, especially when compounded with plasticizers, fillers, fibers, and bioactive additives to suit specific application needs [3].

A key challenge in PHBV research and product development is that biodegradability is not a fixed parameter but an outcome of multiple factors such as environmental conditions (i.e., temperature, moisture, oxygen availability, microbial community, nutrient content), the polymer composition, molecular weight, crystallinity, chemical structure, presence of additives, material format (film, plaque, multilayer, composite), reduction potential, hydrophilicity and breakdown products. However, the extent of the effects from some of these factors remains unclear. Biodegradation is influenced by the susceptibility of the polymer carbon backbone to microbial attack. Research on PHBV biodegradation can be divided into two categories: those that indicate the degree of biodegradation, and those that indicate the mass or weight loss over the study duration. The latter is more prevalent in the literature due to its ease; however, based on ASTM standards, it is not adequate to determine the degree of biodegradability of polymers by themselves. Furthermore, it is the duration of biodegradation that makes complete studies following ASTM standards incredibly rare.

Consequently, laboratory studies often employ standardized test methods to ensure reproducibility and comparability of the results. For instance, ISO 14855 [4] measures the ultimate biodegradability of PHBV in controlled composting environments and determines the extent of biodegradability in terms of CO_2_ evolution, while ASTM D5338 [5] measures biodegradability in aerobic environments under controlled composting at thermophilic temperatures. These test methods are essential to the development of biodegradable plastics such as PHBV and in the standardization of their properties and performances. However, the test conditions, although standardized, can be quite different from the conditions in the “real environment” of use, and the results of the biodegradability of PHBV can differ from the standardized tests.

Indeed, the evidence within the existing literature on PHBV supports the extent of variation across environments and formulations. For example, the neat PHBV material has been found to display low rates of mass loss within soil environments. On the other hand, the incorporation of natural fibers may significantly enhance the degradation rates. This is supported by the different water absorption rates and degradation mechanisms [6]. Similarly, composite materials containing lignocellulosic fillers such as wood flour have shown improved degradation rates when subjected to soil burial tests [7]. For aquatic environments, PHBV films containing phenolic additives such as catechin, ferulic acid, and vanillin were observed to degrade through respirometry analysis. This shows that the degradation kinetics may depend on the medium [8]. Even within composting environments conducted under nominally controlled conditions, disintegration and degradation rates can vary depending on the multilayer structures or the variation in the blend composition, underlining the complexity of extrapolating laboratory results to real-world scenarios [9].

Such a heterogeneous environment poses a practical challenge in lab testing, whereas field testing in a real-world environment is expensive and time-consuming. However, decisions regarding formulation, processing pathways, and end-of-life need to be made in early-stage testing. Hence, there is a significant interest in developing a data-driven approach that can learn relationships between material descriptors, additives, and environmental factors in predicting biodegradation. Recent work within the larger domain of polymer biodegradation has demonstrated the ability of machine learning (ML) methods to predict biodegradation endpoints such as percent biodegradation given input features [10]. This presents a clear path forward for faster testing and hypothesis generation. Beyond polymer-specific datasets, interpretable ML frameworks have been proposed for enhancing prediction and understanding of primary vs. ultimate biodegradation endpoints, highlighting the importance of both prediction and mechanistic insight [11]. Similar work has utilized rank-based learning methods to overcome issues and biases inherent within degradability data, where experimental conditions and reporting are highly varied [12]. ML techniques have also been used simultaneously to predict biodegradation behavior in aquatic environments using meta-analytic datasets that include both material properties and experimental conditions [13].

However, various key unique issues related to the predictive modeling of PHBV biodegradability should be investigated extensively. First, PHBV is often blended with a variety of plasticizers such as citrate esters, natural fibers, and fillers such as cellulose and wood flour, and mineral fillers such as calcium carbonate, and functional additives such as antimicrobial and antioxidants, which can influence water sorption, crystallinity, interfacial microstructure, and microbial degradability [3]. Second, the results of PHBV biodegradability are expressed in diverse forms, such as ultimate biodegradation (CO_2_ evolution), disintegration, fragmentation, mass loss, changes in molecular weight, and surface/morphological changes [14]. Third, scaling up from small laboratory experiments to medium/large laboratory tests, and then to the field, is complicated by non-linear effects, e.g., oxygen transfer, temperature gradients, and moisture heterogeneities, which make it difficult to extrapolate the results from standardized conditions.

In this context, this study presents for the first time a computational framework that automates the process from literature-based data collection to the prediction of weight loss in scl-PHA biopolymers. The model accurately predicts physical disintegration across various experimental scales and environments, including soil, marine, freshwater, and compost settings. Initially, a comprehensive dataset on degradation-related weight and mass loss of PHBV-based formulations was manually curated by systematically collecting, assembling, and harmonizing data on material characteristics, environmental exposure conditions, and biodegradation behavior. The dataset included a combination of numerical and categorical variables. The mixed data types and potential nonlinear relationships motivated the use of advanced ML methods combined with appropriate preprocessing strategies. Subsequently, multiple regression-based QSAR models were developed and rigorously validated for predicting the biodegradation behavior of the investigated formulations. The models achieved high predictive accuracy, indicating robust structure-degradation relationships. However, given the complexity and heterogeneity of polymer structures and degradation processes, the model’s ability to generalize across different material–environment combinations should be further examined. Finally, the most important features governing biodegradation-induced weight loss were identified, and their effect on the predictions was examined. Degradation environment, exposure time, and hydroxybutyrate (HB) ratio were revealed as key factors in weight loss, showing that although weight loss was increased in duration and temperature, soil and polymer conditions (such as high crystallinity) hindered it significantly.

The final predictive model was implemented as a user-friendly web application on the Jaqpot computational platform (https://jaqpot.org/, accessed on 2 April 2026) and is openly accessible to the scientific community through the ANIPH virtual organization.

## 2. Materials and Methods

### 2.1. Workflow of Model Development

**Figure 1:**
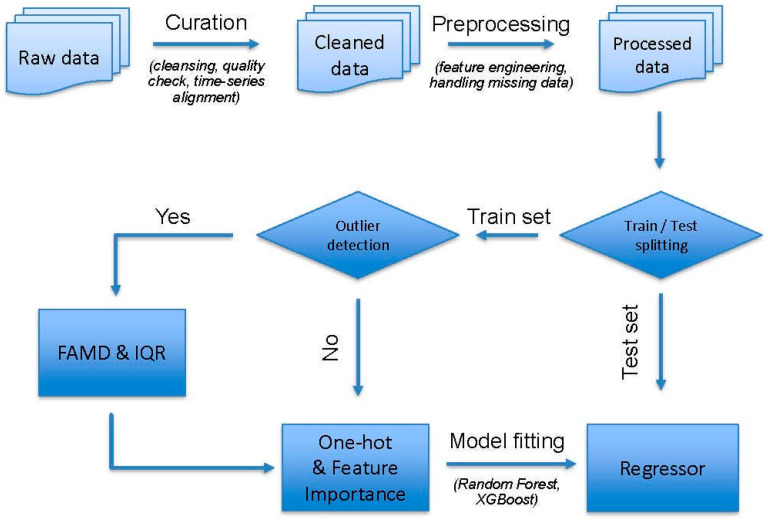
Schematic overview of the data-driven workflow developed in this study.

The overall methodological workflow adopted for this study is presented below in Figure 1 and involves distinct phases of data curation, preprocessing, and data-informed modeling. The raw data on weight loss were collected manually from the literature and underwent an initial curation step, including consistency checking, quality control, and time-series alignment. The next phase involved data preprocessing, including feature engineering, normalization, and handling of missing values, to yield the processed dataset. The processed dataset was split into the training and test sets, followed by outlier detection on the training set. When outliers were detected, factorial analysis of mixed data (FAMD) [15] and interquartile range (IQR) analysis [16] were performed to reduce their effects. One-hot encoding of the categorical variables and feature importance analysis were performed. Finally, the regression models were developed using the ensemble learning algorithms of Random Forest [17] and Extreme Gradient Boosting (XGBoost v. 2.1.3) [18] to predict the weight or mass loss of PHBV-based formulations under different environmental conditions and test scales. Both models are well-suited for polymers informatics applications due to their ability to capture nonlinear relationships and interactions among descriptors. Model performance was evaluated using the coefficient of determination (R^2^), root mean squared error (RMSE), and mean absolute error (MAE) for training, test, and validation sets. To further enhance performance, grid search–based hyperparameter optimization was conducted using cross-validation on the training data. Optimal hyperparameters were selected based on minimizing prediction error, and the final models were retrained before evaluating the test set.

### 2.2. Biodegradation-Induced Weight/Mass Loss Database Construction for PHBV-Based Formulations

The first stage in constructing the biodegradation-induced weight or mass loss database of PHBV-based formulations containing different additives or building blocks was data collection. The quality of the data collected was critical to the overall study. Data were obtained through a comprehensive literature-mining process that included peer-reviewed journal articles, theses, and published experimental studies addressing PHBV degradation under diverse environmental conditions. The selected studies investigated the degradation behavior of the studied formulations in soil, compost, freshwater, and marine environments, considering medium- and large-scale laboratory tests as well as real environmental exposure conditions. Emphasis was placed on identifying studies that reported quantitative degradation metrics based on “weight or mass loss”, used as an indicator of biodegradation-induced disintegration, quantifying the physical loss of material mass over time. These studies also revealed associated environmental parameters and material characteristics of the investigated formulations.

#### 2.2.1. Literature Search Strategy

In accordance with the PRISMA 2020 guidelines [19,20], a literature search was carried out in the Scopus database [21]. The search identified relevant literature on the properties associated with biodegradation-induced weight or mass loss of PHBV-based formulations, including natural and synthetic additives such as plasticizers, fillers, stabilizers, antioxidants, bio-based compounds, and other components.

The literature search used a combination of Boolean operators, with a focus on the Title, Abstract, and Keywords fields (TITLE-ABS-KEY). Three concept blocks were used with the AND operator, and each block was used to identify literature on the following: (1) the copolymer PHBV as the biodegradable material, (2) weight/mass loss properties associated with the biodegradation of the material, (3) a set of additives/components used with the PHBV-based formulations to enhance their biodegradability.

The final search query in the Scopus database was structured as follows:

TITLE-ABS-KEY ((“PHBV” OR “polyhydroxybutyrate-co-valerate” OR “poly(3-hydroxybutyrate-co-3-hydroxyvalerate)”) AND (“biodegradation” OR “decomposition” OR “microbial degradation”) AND (“weight loss” OR “mass loss” OR “weight reduction” OR “mass reduction” OR “disintegration” OR “fragmentation” OR “erosion” OR “biodegradation rate” OR “percentage mass loss” OR “weight loss %”) AND (“lignin” OR “lignins” OR “citric acid” OR “citric ester” OR “acetyl tributyl citrate” OR “ATBC” OR “triacetin” OR “triethyl citrate” OR “TEC” OR “epoxidized soybean oil” OR “epoxidized cottonseed oil” OR “epoxidized natural rubber” OR “ENR” OR “soybean oil” OR “Vish-E filler” OR “starch” OR “starch-based fillers” OR “cornstarch” OR “alginate” OR “alginic acid” OR “pure cellulose” OR “cellulose fibers” OR “wood flour” OR “woodflour” OR “WF” OR “wheat straw fibre” OR “wheat straw fiber” OR “lignocellulosic” OR “miscanthus” OR “olive pomace” OR “propionylated abaca fiber” OR “catechin” OR “ferulic acid” OR “vanillin” OR “polylactic acid” OR “PLA” OR “poly(ε-caprolactone)” OR “PCL” OR “polybutylene adipate-co-terephthalate” OR “PBAT” OR “polyethylene oxide” OR “PEO” OR “flax fibers” OR “flax fibres” OR “calcium carbonate” OR “CaCO3” OR “halloysite” OR “modified halloysite” OR “lignin-coated cellulose nanocrystals” OR “boron nitride” OR “quercetin” OR “DDGS” OR “distillers dried grains with solubles” OR “posidonia oceanica” OR “gallic acid” OR “ammonium quaternary salts” OR “castor oil” OR “limonene” OR “thymol” OR “oregano essential oil” OR “sorbitol” OR “maltodextrin” OR “dicumyl peroxide” OR “Licowax”)).

#### 2.2.2. Study Selection and Screening

The records identified through the database search were further exported for evaluation. Titles and abstracts were initially screened to assess their relevance in relation to the biodegradation of PHBV-based formulations containing diverse additives under laboratory or natural environmental conditions. No restrictions were placed on the publication year to ensure a comprehensive historical record was obtained. Additionally, only articles published in English were included. During the screening phase, review articles, conference papers, editorials, notes, and book chapters were excluded. Full-text articles were subsequently assessed in detail according to pre-defined inclusion and exclusion criteria.

The inclusion criteria comprised studies that:

(i) Investigated PHBV or PHBV-based composites;
(ii) Examined biodegradation, microbial degradation, or disintegration processes under laboratory or real environment conditions;
(iii) Reported quantitative, time-resolved degradation metrics based on weight or mass loss (%);
(iv) Evaluated the incorporation of additives such as fillers, plasticizers, nucleating agents, antioxidants, bio-derived materials, or other functional compounds within the polymer matrix.

The exclusion criteria eliminated studies that examined other types of polymers apart from PHBV, studied degradation mechanisms unrelated to the process of biodegradation or microbial activity, solely employed gas evolution-based mineralization methods (e.g., measurement of evolved CO_2_), which failed to include the measurement of mass or weight loss, or examined the addition of compounds that were not incorporated into the matrix of the polymer.

**Figure 2:**
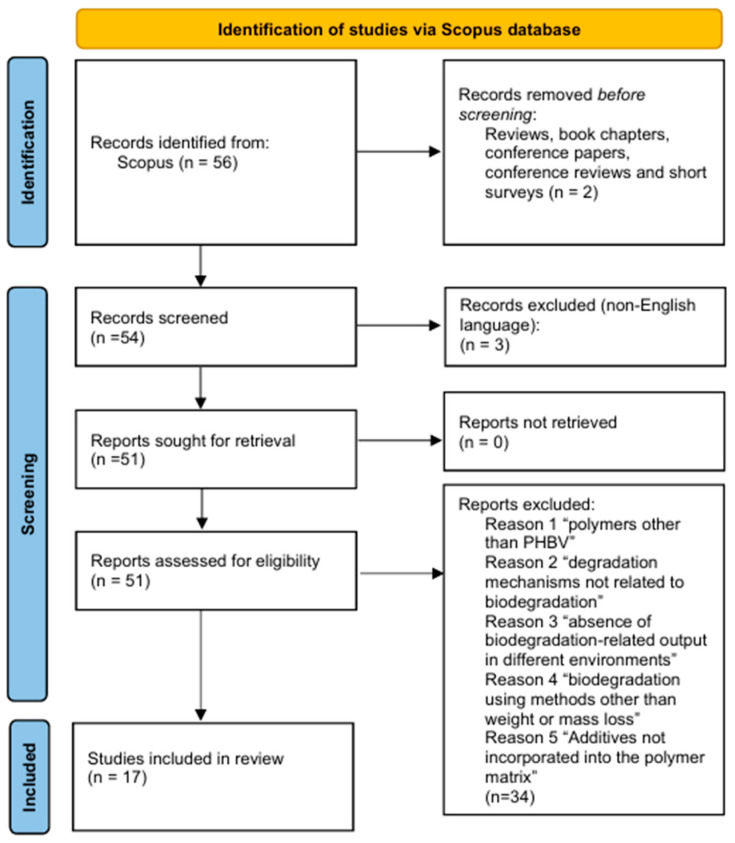
PRISMA 2020 flow diagram [19] illustrating the identification, screening, eligibility, and inclusion of studies investigating the biodegradation-induced weight or mass loss of PHBV-based materials containing natural and synthetic additives.

Initially, 56 publications from 1991 to March 2025 were identified from the Scopus database’s advanced search tool using the predefined search query. After removing duplicates and an initial screening, a full-text evaluation was conducted for all publications, following specific eligibility criteria. Only publications relevant to the specific scope of this study, namely on biodegradation-induced weight or mass loss of PHBV-based formulations containing specific additives, conducted under soil, compost, freshwater, marine, or other environmentally relevant conditions, including medium- to large-scale experiments conducted in the laboratory or under natural environmental conditions, were considered. Further, only publications containing primary experimental data were eligible. Although the exclusion criterion for non-English publications may have resulted in eliminating significant findings from regions where significant research activity was conducted (e.g., Japan and China), this criterion was applied to ensure English was used as a primary language for international scientific communication. Further, only full-text publications were considered to ensure access to all necessary details on the methodologies and experiments used. A total of 17 peer-reviewed publications [6,7,22,23,24,25,26,27,28,29,30,31,32,33,34,35,36] formed the final database used for this study’s analysis and prediction of biodegradation-induced weight or mass loss for PHBV-based formulations containing different additives and building blocks. The specific study selection approach used in this study is illustrated in the PRISMA 2020 flow diagram [19] depicted below in Figure 2.

#### 2.2.3. Data Extraction and Synthesis

For each eligible study, information related to PHBV formulation parameters and physicochemical properties was systematically extracted, including polymer composition, molecular and structural characteristics, and the type and concentration of additives or bio-derived compounds incorporated into the matrix. In addition, details regarding the biodegradation conditions, such as exposure environment, test duration, specimen size or geometry, and experimental setup, were collected with the reported quantitative degradation outcomes based on weight or mass loss (%). These data were used to conduct qualitative analysis to establish trends in biodegradation-induced disintegration behavior associated with different additive categories and environmental conditions.

All available published data, including numeric values, polymer names, additive descriptors, and physicochemical property data, were organized in tabular format (Microsoft Excel files) to facilitate transfer and subsequent analysis. When property data were only available in graphical form, numerical values were manually extracted and curated from figures, plots, and digitized tables reported in the main text using the Plotdigitizer software (version 3) [37].

#### 2.2.4. Data Curation

Following data extraction, curated databases were constructed in a structured spreadsheet format (Microsoft Excel) to systematically capture the multivariate aspects of biodegradation-induced weight or mass loss in PHBV-based systems. The extracted data from all eligible studies were integrated into a unified database composed of five worksheets, each corresponding to a distinct data category, as summarized below in Table 1.

This structured organization ensured that uniform representation of material descriptors, additive properties, environmental exposure conditions, sample characteristics, and quantified degradation results was achieved.

The worksheets were developed to ensure data harmonization and cross-referencing of heterogeneous data collected from different experimental procedures and data representation styles. In addition, a worksheet was created to record all abbreviations, symbols, and definitions used for data variables to ensure proper understanding during subsequent data processing modeling.

Each study was assigned a unique identification number (study ID), and all corresponding data were subsequently entered as individual rows in the database. In situations where a study reported more than one physicochemical property or experimental condition, distinct data instances were created to represent individual conditions. However, all these data instances, which represented varying experimental conditions, still carried the study ID of their respective original study. In total, 17 distinct study IDs were included, corresponding to 226 independent instances in “Worksheet_1_Materials_features” and 133 independent instances in “Worksheet_2_Environmental_features”, “Worksheet_3_Biodegradation_features”, and Worksheet_4_Time_point_features”, collectively yielding 1546 time-resolved weight or mass loss observations. This structure enabled both within-study and between-study analyses, allowing variability to be examined at the instance level as well. In Table 2, we summarize the distribution of instances and data pairs (time point, weight, or mass loss percentage) per study. Note that the study IDs were non-sequential as they were extracted from a larger data library.

Table A1, Table A2, Table A3 and Table A4 in Appendix A outline a comprehensive summary of all input (feature) and output (target) variables used to generate the PHBV-based formulations’ biodegradation-induced weight or mass loss data library. Table A1 lists 46 materials descriptors (23 categorical & 23 numerical) associated with PHBV-based formulations’ composition, additives, molecular and physicochemical properties, morphological characteristics, and surface characteristics. Table A2 outlines 29 environmental features categorized into 17 numerical and 9 categorical variables and describing a range of biochemical environment variables, physical medium, temperature, pH, moisture, salinity, nutrient composition, solids composition, and standardized testing protocols.

Table A3 details the biodegradation-induced weight or mass loss response variables included, and Table A4 summarizes the degradation time-series output variables. Each variable is described based on its definition, measurement unit, range of values, and type (categorical or numerical). The “Worksheet_3_Biodegradation_features” and “Worksheet_4_Time_points_features” datasets were organized in a hierarchical format comprising multiple study- and instance-level observations, and representing repeated measurements recorded under the same experimental conditions, see, for example, the first five rows in Table 3 and Table 4, respectively. Note that not all instances have the same number of data points.

A comprehensive raw data library for PHBV biodegradation-induced weight or mass loss, including all variables across the four categories, is available in the AUA Zenodo repository [38].

Due to manual data extraction from heterogeneous literature sources, some additional noise may be present in the dataset. This was mitigated through iterative quality checks using descriptive statistics. Thus, model predictions should be interpreted in terms of long-term degradation trends rather than short-term ones, where high numerical precision is required.

#### 2.2.5. Data Pre-Processing

To facilitate statistical analysis, the dataset “Worksheet_3_Biodegradation_features” was transformed into a long format. As a result, each weight loss value was *melted* into a separate row, such that every row represented a single weight loss observation associated with its corresponding “Study_id”, “Instance”, and “Sample_name”. This restructuring preserved the hierarchical relationships in the data while enabling instance-level analysis of weight loss outcomes.

The resulting dataset was combined with the corresponding melted “Worksheet_4_Time_points_features” dataset. This integration aligned each weight loss observation with its associated time point, while retaining the study-, instance-, and sample-level identifiers. The final merged “t_y_values” dataset comprised 1546 rows and 5 columns, with each row representing a single degradation time–weight loss % observation pair.

The melted and merged “t_y_values” dataset was subsequently integrated with the “Properties” dataset, which arose from merging the “Worksheet_1_Materials_features” and “Worksheet_2_Environmental_features” datasets. This final merge enriched each observation with the corresponding physicochemical and environmental properties, resulting in a comprehensive dataset comprising 20 columns, as summarized in Table 5 below. Features with more than 80% missing values were removed.

The final merged dataset consisted of two identifier columns (“Study_id” and “Instance”), one target variable, “Weight_loss_%”, and 17 feature columns. Of the feature variables, seven were numerical, and ten were categorical, capturing a diverse set of formulation, material, and experimental characteristics. This combination of variable types enabled comprehensive modeling of weight loss behavior.

To reduce sparsity and improve interpretability, categorical features with many small or semantically similar categories were consolidated. For instance, the “Degradation_Environment” feature originally contained 12 categories, some with very few observations (e.g., “vermicompost” with 4 instances). These categories were mapped into four broader, domain-relevant groups: Soil, Marine/Aquatic, Compost/Organic Fertilizer, and Laboratory/Mineral Media. This approach preserved experimental context while simplifying the dataset to facilitate visualization, modeling, and interpretation. Table 6 illustrates the mapping of original to merged categories for “Degradation_Environment”. The same procedure was applied to the features: “Degradation_mechanism”, “Sample_shape/Morphology”, “PHA_degrading_microbes”, and “Additive_type_1”. In Table 7, a summary of all features affected by this categorical dimensional reduction is detailed.

Following the categorical consolidation, missing values (18.3%) in the numerical feature “T_deg” (degradation temperature) were addressed using a targeted imputation strategy. Since degradation temperature was primarily determined by the degradation environment, missing “T_deg” values were filled using the mean temperature of comparable conditions from the same study id. For example, missing values of a given study id in the “Marine/Aquatic” environment were imputed using the mean temperature from all other instances in the same environment. This approach preserved the environmental context of each observation while ensuring that the dataset remained complete for subsequent analysis.

**Figure 3:**
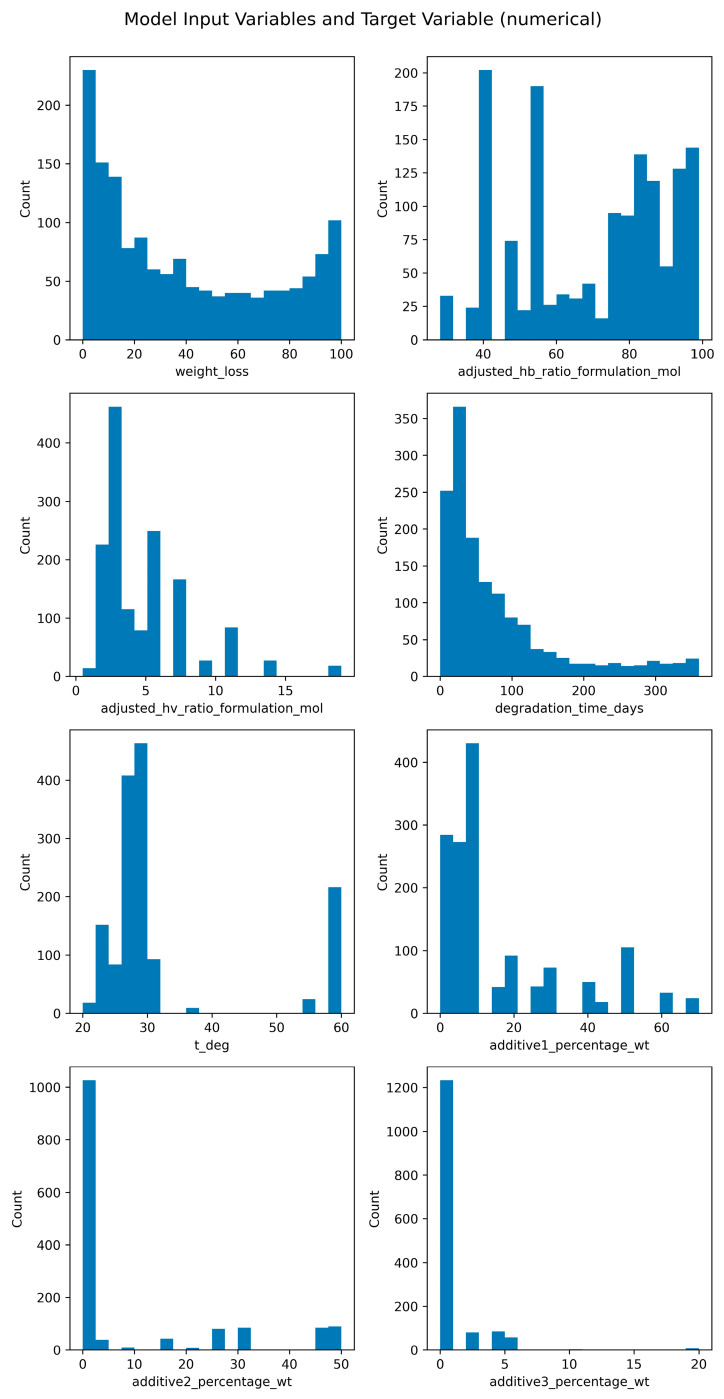
Distribution of model input variables: the target value (top left) and numerical features.

**Figure 4:**
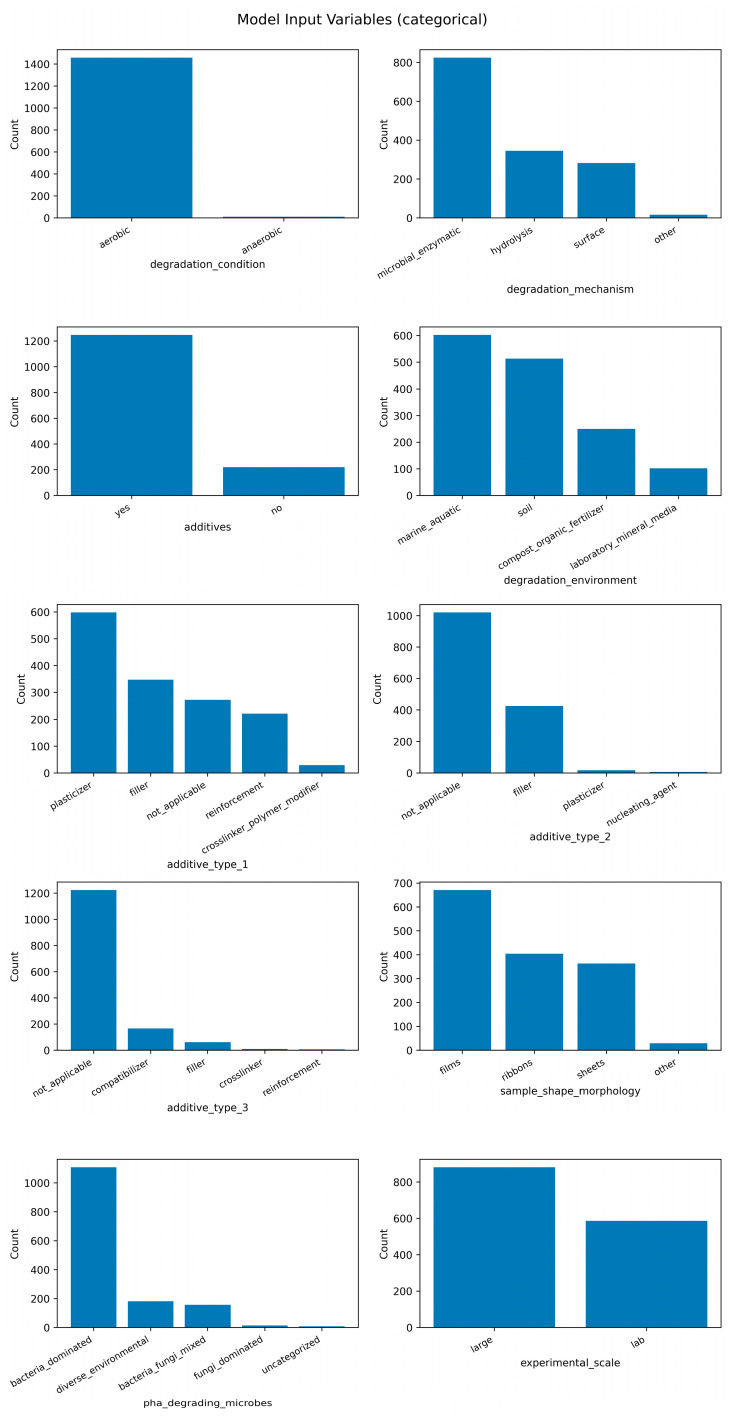
Distribution of model input categorical variables.

Finally, rows containing missing values in any of the feature columns were excluded, resulting in a final dataset of 1467 complete observations. The distributions of all features in the final dataset are summarized in two figures: Figure 3 shows the distributions of numerical features, while Figure 4 presents the distributions of categorical features, highlighting the range and skewness of each variable.

### 2.3. Biodegradation QSAR Model Development and Validation

The dataset was partitioned into training and testing subsets to enable robust and unbiased evaluation of model performance. The splitting was performed before feature importance-based selection, where dimensionality reduction via Factor Analysis of Mixed Data (FAMD) and outlier removal ensured that the test set remained unseen during feature encoding. The train-test split was performed at the instance level, allowing time and weight loss data from the same instance to appear in both sets.

**Figure 5:**
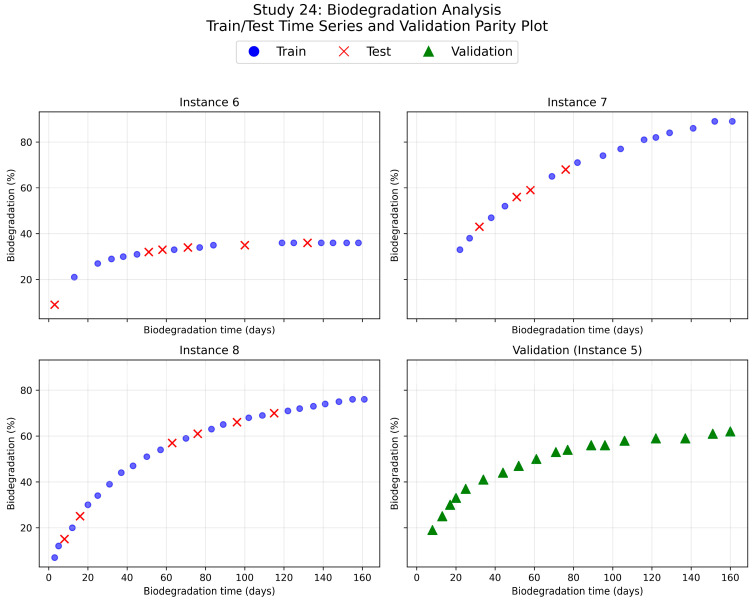
Train/test splitting and held-out data points (validation set) of the Study id 24.

To rigorously evaluate model performance on held-out data, entire instances were excluded from training and testing. From study IDs with more than two instances, one instance with more than five data points was randomly selected and reserved in a validation set. This approach enabled assessment of the model’s ability to predict polymer weight or mass loss behavior across different properties. Figure 5 shows a representative example for Study id = 24, which consists of 4 instances. Nevertheless, as the held-out instances were derived from the same experimental studies, some correlation related to shared properties and measurement conditions may remain, and the validation results should therefore be interpreted as an initial indication of model generalization rather than a fully independent assessment.

The size of the unseen (validation) set is 167 (11.4%). Then, 20% (260) of the remaining observations were held out as the test set, while the remaining 80% (1040) were used for training.

FAMD was used to find the most informative features. This method is suitable for datasets containing both numerical and categorical variables, as it simultaneously captures variance in continuous features and associations among categorical features. We selected fewer components for the final transformation, keeping only components with eigenvalues above 1%, ensuring that each retained component contributed meaningfully to explaining the overall variance. The original feature matrix was transformed into a lower-dimensional representation, preserving the essential structure of the data and facilitating subsequent modeling and analysis.

To determine the influence of extreme values on subsequent analyses, we employed an outlier detection procedure based on the interquartile range (IQR) for each FAMD component. Observations falling outside the range [Q_1_ − α IQR, Q_3_ + α IQR] were flagged as outliers and excluded from the dataset. The effect of this procedure will be discussed in the following section, where we demonstrate that polymer informatics models benefit from data diversity rather than aggressive statistical filtering.

After removing the outliers, categorical features were further encoded using one-hot encoding to enable compatibility with tree-based learning algorithms. Feature importance analysis was also performed using a Random Forest (RF)–based model to reduce dimensionality and improve interpretability. Features exhibiting positive (importance) values were retained for further modeling, while features with negligible contributions were excluded. Thus, this importance-driven feature selection reduced the complexity of the model and prevented overfitting, while preserving the most relevant variables to polymer weight loss behavior.

### 2.4. Model Explanation & Information Extraction

**Figure 6:**
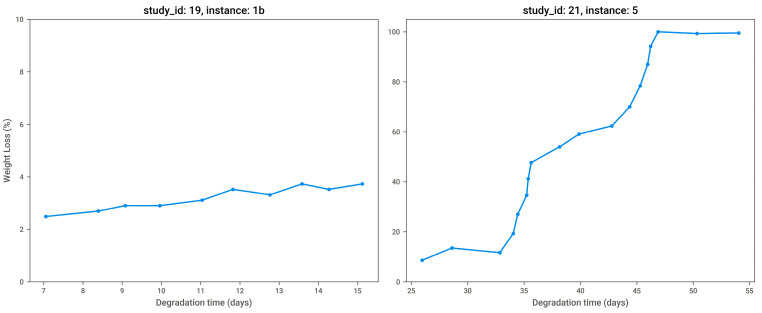
Weight loss percentage over time for two study instances.

**Figure 7:**
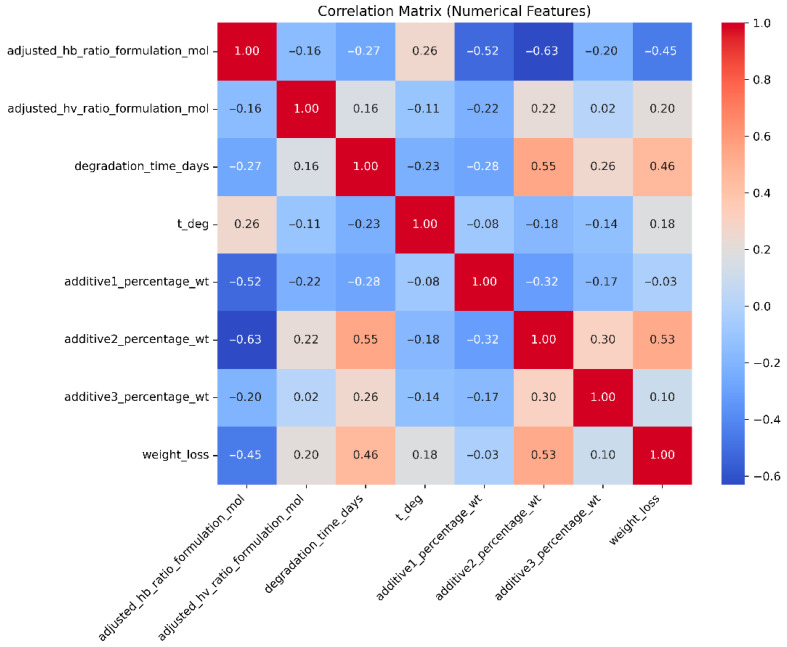
Correlation matrix showing pairwise relationships among numerical features.

We were interested in predicting the biodegradation-induced weight or mass loss percentage (%) based on the degradation time point (in days) and polymer properties. However, it was important to highlight that weight loss did not increase monotonically over time (Figure 6). While the correlation matrix (see Figure 7) confirmed that degradation time and weight or mass loss were positively correlated (0.46), the relationship was moderate rather than strong, indicating the influence of additional factors and justifying the use of a multivariate modeling approach.

The complete processed dataset used for analysis and modelling is provided in https://github.com/FSL-AUA/Weight-loss-model.git, accessed on 2 April 2026.

RF and XGBoost models were initially trained and evaluated using the complete dataset without outlier exclusion to evaluate the robustness of the proposed polymers informatics framework. The final dataset consisted of 43 selected descriptors, with 1040 samples in the training set and 260 samples in the test set.

Model performance was evaluated using:

- **R^2^**, which measures the proportion of variance in the observed data, was explained by the model:

$$R^{2}=1-\frac{\sum_{i=1}^{n}{\left(y_{i}-{\hat{y}}_{i}\right)}^{2}}{\sum_{i=1}^{n()}{\left(y_{i}-\bar{y}\right)}^{2}}$$

where $y_{i}$ is the true value, ${\hat{y}}_{i}$ is the predicted value, and $\bar{y}$ The mean of the true values.

- **MAE** defined by

$$MAE=\frac{1}{n}\sum_{i=1}^{n}\left|y_{i}-{\hat{y}}_{i}\right|$$

- **RMSE** quantifies the average magnitude of prediction errors:

$$RMSE=\sqrt{\frac{1}{n}\sum_{i=1}^{n}{\left(y_{i}-{\hat{y}}_{i}\right)}^{2}}$$

## 3. Results & Discussion

### 3.1. Dataset Construction

Literature data mining of 17 peer-reviewed research studies was undertaken to manually curate a structured dataset on the biodegradation-induced weight or mass loss of PHBV biopolymers formulated with a range of additives and building blocks. The curated raw dataset used for the data pre-processing step is publicly available in the associated data repository (AUA Zenodo repository) [38]. An overview of the compositional, molecular, physicochemical, environmental descriptors, and biodegradation outcomes of the PHBV-based formulations assessed across the multiple cited studies is presented in Table A1, Table A2, Table A3 and Table A4 in Appendix A. The biodegradation endpoint was precisely defined in line with standardized biodegradability assessment practices and based on biodegradation-induced disintegration measured as time-series data and expressed as “weight/mass loss percentage”. Degradation performance metrics were obtained directly from published weight/mass loss percentage–time curves, extracted from the literature using the digitization of graphical data.

### 3.2. Performance of QSAR-Based Degradation Models

RF and XGBoost models effectively predicted polymer weight loss, achieving test coefficients of determination (R^2^) values above 0.92. The predictive performances of the models are summarized in Table 8. Both models achieved high accuracy on the training dataset, with R^2^ exceeding 0.96. While test R^2^ values remained strong, the corresponding RMSE and MAE indicated moderate prediction errors on individual observations. These error metrics reflected the inherent variability in polymer degradation data and showed that, although the models effectively captured overall trends, some deviations remained at specific time points or under less common environmental conditions. Overall, the relatively low MAE compared to the typical range of weight loss values indicated that predictions were generally reliable for practical applications, especially in assessing long-term degradation behavior.

Grid search–based hyperparameter optimization was performed to enhance the predictive performance of both models. This procedure systematically evaluated a predefined range of hyperparameter combinations, such as the number of trees, maximum tree depth, learning rate, and minimum samples per leaf, using cross-validation on the training dataset. By assessing model performance across these configurations, the grid search identified the combination of hyperparameters that minimized prediction error and improved generalization to unseen data. The optimal hyperparameters selected for each model, which guided the final model training, are summarized in Table 9.

The tuned models were evaluated using cross-validation (5-fold) and test datasets, as summarized in Table 10. Both tuned models showed improved generalization compared to the baseline configurations, with reduced overfitting as evidenced by the smaller discrepancy between training and test R^2^ values.

**Figure 8:**
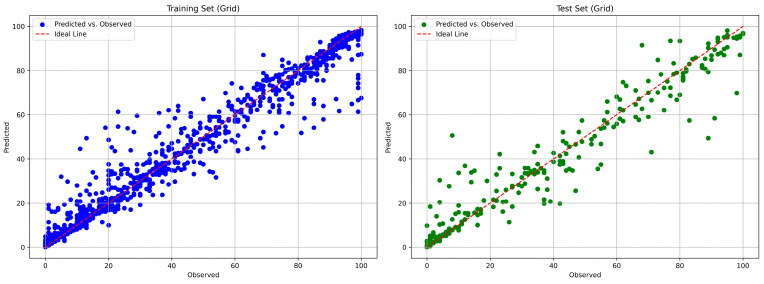
RF: Comparison of predicted and true weight loss percentages for the training set (**left**) and the test set (**right**).

**Figure 9:**
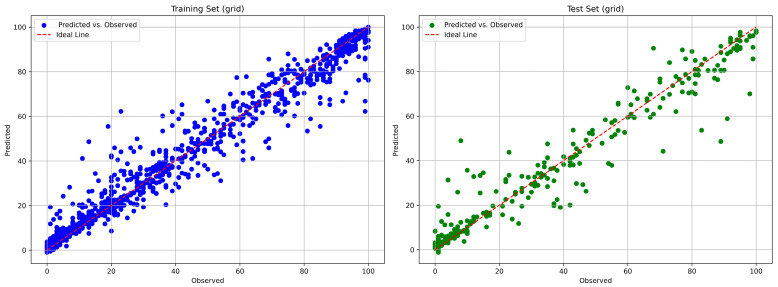
XGBoost: Comparison of predicted and true weight loss percentages for the training set (**left**) and the test set (**right**).

The comparisons of the predicted and the true weight or mass loss percentages for the tuned models are presented in Figure 8 and Figure 9 for the RF and the XGBoost model, respectively.

**Figure 10:**
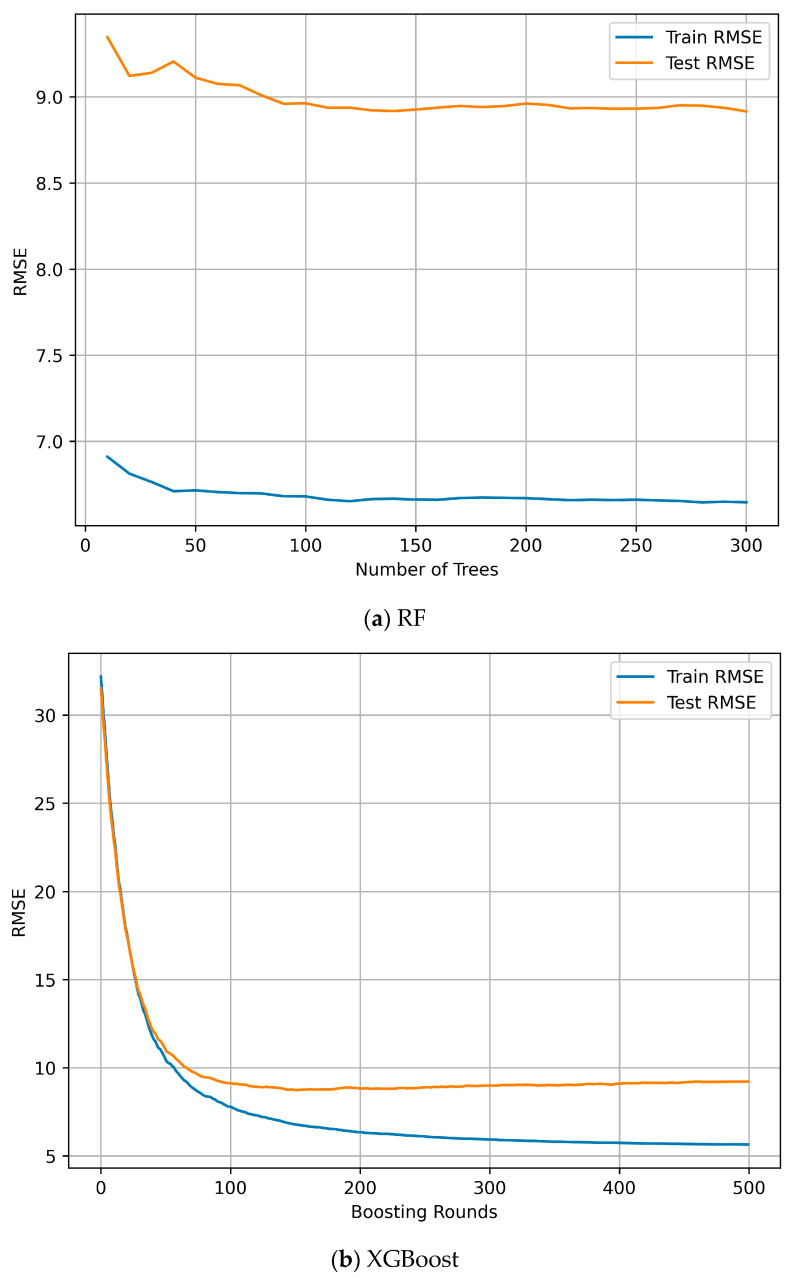
The convergence behavior of the RMSE for the training and test sets. For the RF, the RMSE was represented as a function of the number of trees (**a**) and as a function of the number of estimators for the XGBoost model (**b**).

In Figure 10a, the convergence of the RMSE as a function of the number of trees is also presented. Training RMSE decreased with increasing number of trees, while test RMSE quickly stabilized, indicating a stable generalization behavior of the RF model. The convergence of the RMSE with respect to the number of estimators is shown in Figure 10b. The evolution of training and test RMSE with increasing boosting rounds for the XGBoost model exhibited early convergence of test performance.

To evaluate whether the imputation of the degradation temperature (t_deg) introduced bias into the model, a sensitivity analysis was conducted by retraining the models without this feature while keeping the dataset and optimized hyperparameters unchanged. The resulting performance metrics were compared with those obtained using the imputed dataset (Table 11). Only minor decreases in predictive performance were observed (approximately 0.01 in test R^2^ for both models), indicating that the targeted imputation strategy did not substantially bias the models toward average environmental conditions.

**Figure 11:**
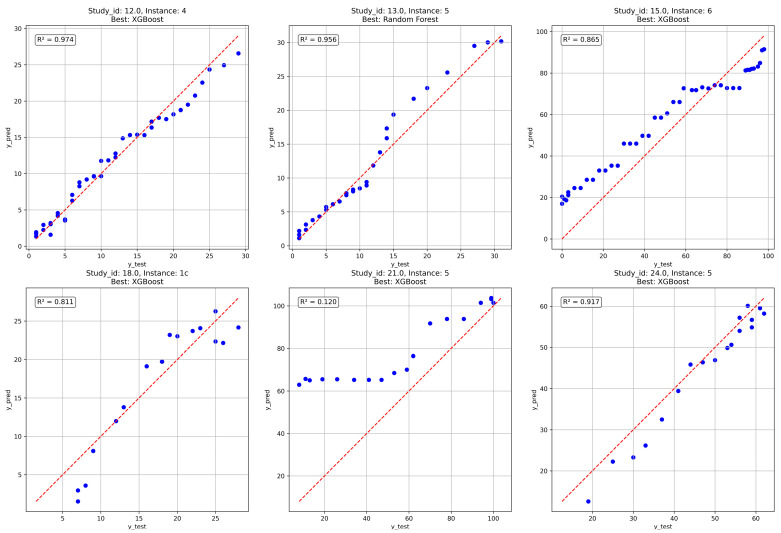
Comparison of predicted and true weight loss (%) of fully unseen instances. The dashed red line represents perfect agreement (y = x).

The tuned models were evaluated on an independent unseen dataset (validation set) not used during training or hyperparameter optimization to further assess model robustness, see Figure 5. The resulting R^2^ scores are reported in Table 12. The XGBoost model retained strong predictive performance on the unseen dataset, whereas the RF model exhibited a substantial decrease in R^2^. This result indicated that XGBoost provided superior robustness to dataset shifts and experimental variability when outliers were not excluded. The best-performing estimated time series from each model are presented in Figure 11, along with their corresponding R^2^ scores. Overall, five out of six cases were estimated accurately. For the remaining case that performed poorly, the predictions were, however, accurate for longer degradation times. This was particularly important because accurately estimating long degradation times was critical to correctly classifying a polymer as biodegradable or not. These results further highlight that the model performs best in capturing general degradation trends, whereas precise quantitative predictions may be less reliable for formulations that are underrepresented in the training data or for shorter degradation times.

### 3.3. SHAP-Based Interpretation of Degradation Models

**Figure 12:**
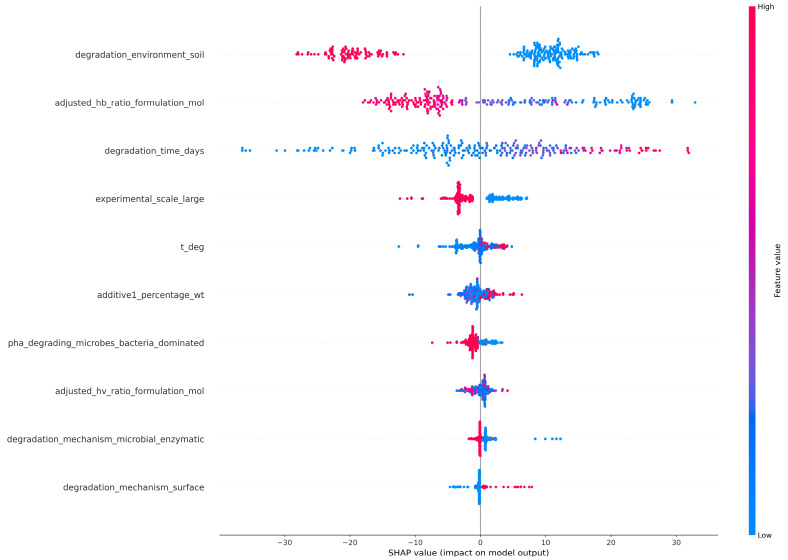
Top 10 feature contributions of the RF model using SHAP values.

SHapley Additive exPlanations (SHAP) values were applied to quantify feature importance by measuring the contribution of each input variable to the model’s predictions. The SHAP summary plot provided a global overview of how different features impacted an ML model’s output across the entire dataset. It should be noted that SHAP values describe the contribution of variables to model predictions and therefore reflect statistical associations within the dataset rather than causal relationships governing biodegradation mechanisms. The SHAP summary plot for the RF model (Figure 12) indicated that biodegradation-induced weight or mass loss was primarily governed by the “degradation environment”, and soil environment emerged as the most influential feature. Observations corresponding to soil conditions predominantly exhibited negative SHAP values, indicating reduced predicted weight loss relative to other environments. This reflected the restrictive nature of soil systems, where limited oxygen diffusion, heterogeneous moisture distribution, and variable microbial accessibility constrained degradation processes. Beyond environmental effects, polymer composition also played a critical role, with higher HB content consistently associated with lower predicted weight loss, likely due to increased crystallinity and reduced enzymatic accessibility. Degradation time exhibited a clear positive contribution, although its impact was secondary to environmental constraints in the RF model. Additional features, including “experimental scale”, “degradation temperature”, “additive content” and “microbial dominance” revealed moderate contributions, whereas “degradation mechanisms” exhibited the lowest contribution.

**Figure 13:**
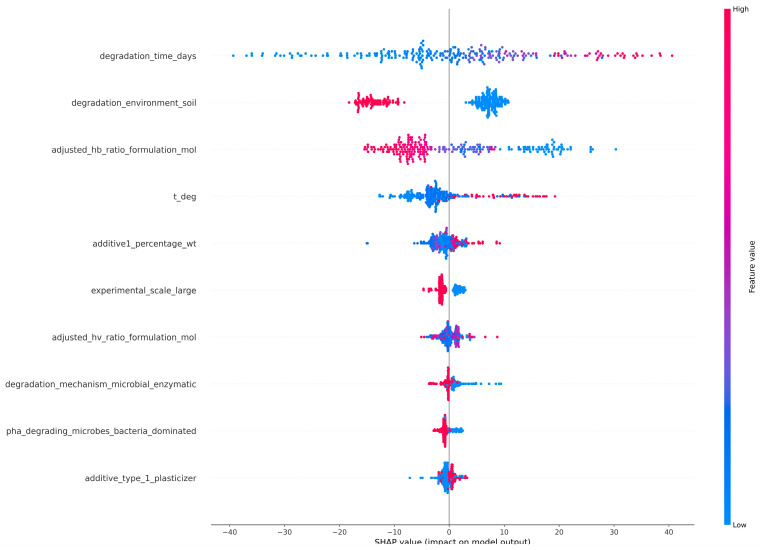
Top 10 feature contributions of the XGBoost model using SHAP values.

On the contrary, the feature importance plot of the SHAP analysis of the XGBoost model (Figure 13) indicated that the primary factor affecting the weight/mass loss was the “degradation time”. Short degradation times were associated with strongly negative SHAP values, while longer exposure periods increased the predicted mass loss, reflecting cumulative degradation processes and potential acceleration phases. Although the “degradation environment” remained a highly important factor, it ranked lower than degradation time in the XGBoost model. Other variables, including “temperature”, “additive concentration”, “polymer composition”, exhibited more pronounced effects compared to the RF model, indicating differences in feature utilization between the two modeling approaches.

The difference in top-ranked features between the two models was attributed to the distinct ways in which RF and XGBoost handle feature interactions and data structure. RF, relying on bagged decision trees, tends to highlight features that consistently reduce impurity across many trees, favoring variables with strong marginal effects such as categorical environment types. XGBoost, on the other hand, builds trees sequentially with gradient boosting, emphasizing features that help correct residual errors from previous iterations. This enables XGBoost to capture temporal effects and subtle nonlinear trends, making degradation time a more influential factor in its predictions. Overall, these results suggest that environmental conditions play a crucial role in determining the possibility of biodegradation, while time-dependent and formulation-specific factors regulate the progression and kinetics of biodegradation. It should be noted that these findings reflect statistical associations captured by the model rather than direct causal mechanisms controlling biodegradation.

**Figure 14:**
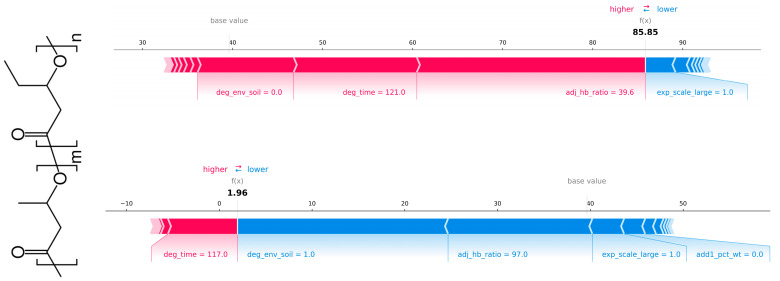
SHAP force plot explaining the RF regression prediction of weight loss (%) for a single test observation: study_id = 18, instance = 2b (**top**) and study_id = 12, instance = 1 (**bottom**), showing positive (red) and negative (blue) feature contributions relative to the model’s base value.

**Figure 15:**
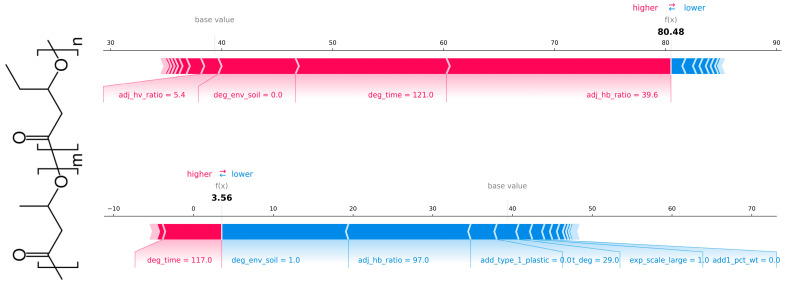
SHAP force plot explaining the XGBoost regression prediction of weight loss (%) for a single test observation: study_id = 18, instance = 2b (**top**) and study_id = 12, instance = 1 (**bottom**), showing positive (red) and negative (blue) feature contributions relative to the model’s base value.

Figure 14, Figure 15 show the SHAP force plot explaining the RF and XGBoost regression prediction, respectively. The prediction of weight loss (%) for an individual test observation was illustrated by decomposing the model output into feature-level contributions. The prediction was constructed by starting from the model’s expected value (base value) and sequentially adding the contributions of each input feature. In the force plot, features shown in red pushed the prediction toward higher predicted weight loss, while features shown in blue pushed it toward lower predicted weight loss, with the length of each bar representing the magnitude of its contribution. The differing effects of features across test instances were clearly shown. For example, the feature “degradation_enviroment_soil” in the top case forced a higher predicted value, whereas in the bottom case, the opposite.

### 3.4. Key Features Influencing PHBV Weight Loss

The study revealed that biodegradation-induced weight loss of PHBV-based formulation was primarily governed by a combination of environmental, temporal, and material-specific descriptors that are summarized below in Table 13.

#### Model-Specific Prioritization

It is noteworthy that both ML models used in this work ranked the features in Table 13 in order of importance, with minor differences:

- For the RF model: The soil environment was ranked first, followed by the adjusted H/B ratio.
- For the XGBoost model: Degradation time was ranked first, followed by the soil environment.

Despite minor differences in ranking between the two ML models, it is apparent that both models consistently ranked environmental and temporal descriptors as the dominant factors in determining the physical degradation (weight loss) of PHBV materials. These results, being specific to the experimental dataset, indicated that the predictions were better interpreted as representations of broader trends rather than accurate quantitative forecasts under varying conditions outside the DOA of this framework.

### 3.5. Effect of Outlier Exclusion

To examine the influence of outlier exclusion on model robustness, the performance of the tuned RF and XGBoost models was compared for datasets with and without statistical outlier removal. 36 samples out of 1040 (3.5%) were excluded from the training set based on distribution-based criteria (see Section 2).

As summarized above in Table 14, outlier exclusion modestly increased cross-validation (CV) mean R^2^ and significantly reduced CV standard deviation (SD) for both models, indicating improved internal stability and reduced variance across folds. However, this increased stability did not consistently translate to improved predictive performance on test data. In particular, test set R^2^ values decreased after outlier exclusion for both RF (from 0.930 to 0.911) and XGBoost (from 0.931 to 0.897), suggesting a loss of generalization when statistically extreme observations were removed.

Evaluation on the independent unseen dataset revealed a subtle effect. Both models demonstrated a clearer improvement, maintaining strong generalization in both cases. Nevertheless, the overall reduction in test set performance indicated that many observations identified as statistical outliers likely corresponded to meaningful polymer degradation mechanisms rather than experimental noise. Given the intrinsic heterogeneity of polymer degradation processes, excluding such samples could reduce the diversity of the training data and limit the model’s ability to generalize across different material–environment combinations. Similar observations were reported in previous polymer informatics studies [39,40].

**Figure 16:**
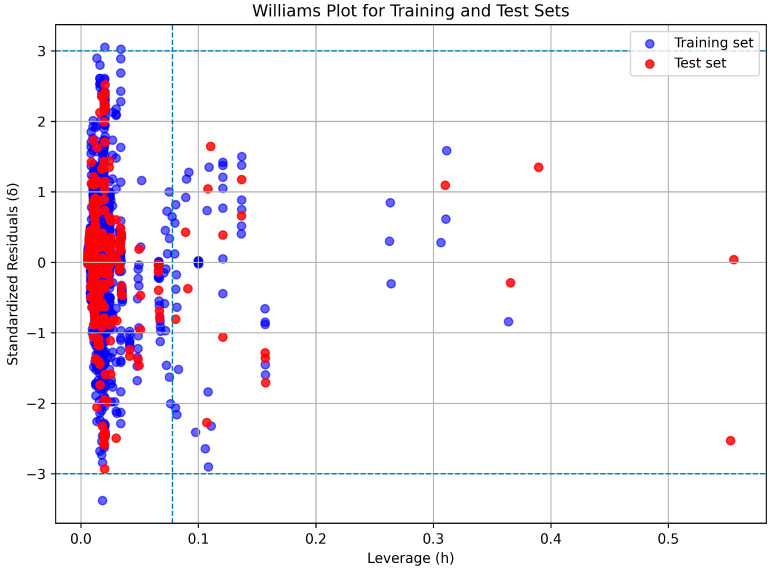
William’s plot showing standardized residuals (δ) versus leverage values (h) for the training (blue) and test (red) datasets. The dashed horizontal lines represented δ = ±3, and the vertical dashed line indicated the warning leverage threshold (h* = 0.0894).

The presence of outliers was further examined using a Williams plot [41], in which standardized residuals (δ) were plotted against leverage values (h). The leverage threshold was defined as h = 3(p + 1)/n, where p is the number of model parameters, and n is the number of samples. Samples with |δ| > 3 or h > h* were considered potential outliers or influential points. Although some samples exhibited leverage values exceeding the warning threshold (Figure 16), their standardized residuals remained within acceptable limits, indicating reliable predictions and confirming the robustness of the model within an extended applicability domain (AD). In Table A5 of Appendix A, the ranges of the input numerical features are reported, defining the model’s AD.

### 3.6. Web Implementation of the Model

The source code for developing the model is publicly available at: https://github.com/FSL-AUA/Weight-loss-model.git, accessed on 2 April 2026. The model has been implemented as a web service on the Jaqpot 5 modelling platform (https://app.jaqpot.org/, accessed on 2 April 2026) and is publicly available (following free registration) at the following URL: https://app.jaqpot.org/dashboard/models/2343/description (accessed on 27 January 2026). Before deployment, the model was evaluated using comprehensive AD analysis to ensure new input samples belong to the descriptor space covered by the training data. The Leverage and Bounding Box methods were applied. Thus, the user can verify if the selected values lie within the AD.

## 4. Conclusions

This study presented an ML framework for predicting biodegradation-induced weight (mass) loss behavior of PHBV-based formulations with different additives as a function of degradation time, using a variety of heterogeneous descriptors that capture material composition, environmental conditions, and experimental parameters. A heterogeneous dataset, comprising repeated measurements from 17 independent studies, was systematically preprocessed and transformed to maintain its hierarchical structure and support data analysis at the instance level. This was achieved through a series of data operations, including categorical consolidation, conditional mean imputation based on degradation environment, and FAMD, which enabled robust and meaningful management of mixed numerical and categorical data.

Using the engineered dataset, both ML models, i.e., RF and XGBoost, showed excellent predictive performance for polymer biodegradation-induced weight loss (%). Without outlier exclusion, tuned RF and XGBoost achieved high test coefficients of determination (R^2^ = 0.930), indicating that over 93% of the variance in experimental weight-loss data could be explained by the selected descriptors. Cross-validation further confirmed model reliability, with mean CV R^2^ values of 0.887 (SD = 0.039) for RF and 0.884 (SD = 0.040) for XGBoost.

When evaluated on an unseen dataset, XGBoost exhibited superior robustness and generalization capability compared to RF. Specifically, XGBoost achieved an unseen R^2^ of 0.829, whereas RF achieved a lower unseen R^2^ of 0.716. This indicated that XGBoost outperformed the RF model and can be used as a reliable tool for predicting polymer degradation, while keeping in mind the domain of applicability imposed by the structure of the dataset used in this study.

Additionally, feature importance analysis showed model-specific prioritization of degradation drivers. In RF, soil environment was the most important feature, followed by adjusted H/B ratio. In contrast, the XGBoost model emphasized degradation time, followed by soil environment. Overall, both models consistently identified temporal and environmental descriptors as the dominant factors.

Outlier analysis showed that statistically extreme data points often corresponded to meaningful polymer degradation mechanisms, rather than experimental noise. Although outlier exclusion improved internal model stability—evidenced by reduced cross-validation SD for both RF (from 0.039 to 0.015) and XGBoost (from 0.040 to 0.014)—it resulted in decreased test-set performance (RF: R^2^ = 0.911; XGBoost: R^2^ = 0.897). In contrast, modest improvements were observed in unseen-dataset performance after outlier exclusion (RF: R^2^ increased to 0.741; XGBoost to 0.856), indicating a trade-off between internal stability and predictive accuracy.

This study provided a reliable data-driven framework that can be used to predict PHBV-based biodegradable materials’ degradation behaviour and indicated that ML models can be used as a reliable tool for assessing their long-term degradation and biodegradability. However, the predictive capability of the proposed models is inherently constrained by the variability and heterogeneity of the literature-derived data, and extrapolation to real-world environmental conditions should be approached with caution. Although the current ML framework has a high degree of predictive accuracy for the phenomenon of weight loss caused by degradation, it is noteworthy that the aforementioned parameter is indicative of physical disintegration rather than complete mineralization. Weight loss is indicative of the empirical loss of physical mass and is therefore a precursor but not a definitive measure of complete conversion to CO_2_ and biomass. Future versions of the aforementioned framework should take into consideration the possibility of the formation of microplastics during the course of disintegration. Aligning predictive frameworks with ultimate biodegradation standards (such as CO_2_ evolution) remains essential to ensure that biodegradable claims meet rigorous environmental safety requirements and do not result in the persistence of persistent micro-scale polymer fragments in the environment.

In future work, the data-driven polymers informatics framework can be utilized on a larger and more heterogeneous dataset, including additional physically meaningful descriptors such as polymer crystallinity. We also plan to expand the dataset by including new experimental data, which will allow further validation of the predictive robustness of the QSAR models. Similarly, other ML models can be used to improve predictive accuracy and generalization.

## Figures and Tables

**Table 1:** Each of the five worksheets represented a distinct category of data within the PHBV-based biodegradation-induced weight or mass loss data library.

Worksheets	Parameters
Worksheet_1_Materials_features	Composition details, Molecular weight distributions (Mw and Mn), Additive presence, Respective additive concentrations, etc.
Worksheet_2_Environmental_features	Temperature, pH, Moisture levels, Oxygen availability, Microbial activity, etc.
Worksheet_3_Biodegradation_features	Weight or mass loss percentages
Worksheet_4_Time_points_features	Degradation time points
Worksheet_5_Metadata	Study_id, Title, Doi

**Table 2:** Distribution of Instances and data pairs (time point, weight or mass loss percentage) across studies.

Study Id	No. of Instances	No. of Data Pairs	Reference
4	2	14	[22]
5	48	192	[23]
6	3	14	[7]
8	1	6	[24]
9	3	24	[25]
10	1	9	[26]
11	2	6	[27]
12	6	253	[28]
13	5	142	[29]
14	4	77	[6]
15	3	126	[30]
16	12	12	[31]
18	20	404	[32]
19	8	93	[33]
20	6	20	[34]
21	5	70	[35]
24	4	84	[36]
Total	133	1546	

**Table 3:** The first 5 rows of the “Worksheet_3_Biodegradation_features” dataset.

Study_Id	Instance	Sample_Name	Weight_Loss %							
4	5	PCA20	2.69511	4.61221	6.68475	9.5863	11.76247	13.47231	22.90236	
4	6	PCA	0.67438	1.34796	2.12516	3.88682	6.06299	6.73656	10.20806	
5	1	PHBV/SOIL	0.57803	4.62428	9.24855	10.4046				
5	2	PHBV/STARCH/SOIL	0.86705	5.78035	10.4046	12.4277				
5	3	PHBV/STARCH/SOIL	2.60116	8.09249	13.8728	15.8959				

**Table 4:** The first 5 rows of the “Worksheet_4_Time_points_features” dataset.

Study_Id	Instance	Sample_Name	Degradation_Time (Days)							
4	5	PCA20	30	60	90	150	240	300	360	
4	6	PCA	30	60	90	150	240	300	360	
5	1	PHBV/SOIL	7	14	21	28				
5	2	PHBV/STARCH/SOIL	7	14	21	28				
5	3	PHBV/STARCH/SOIL	7	14	21	28				

**Table 5:** Data type and completeness summary of the merged dataset.

Column	Completeness	Dtype
Study_id	100.0%	int64
Instance	100.0%	int64
Degradation_time_(days)	100.0%	float64
Weight_loss_%	100.0%	float64
Adjusted_HB_ratio_formulation (mol%)	99.6%	float64
Adjusted_HV_ratio_formulation (mol%)	99.6%	float64
Degradation_condition	100.0%	object
Degradation_mechanism	100.0%	object
Additives	100.0%	object
T_deg	81.7%	float64
Degradation_Environment	100.0%	object
Additive_type_1	100.0%	object
Additive1_percentage (wt%)	95.3%	float64
Additive_type_2	100.0%	object
Additive2_percentage (wt%)	98.1%	float64
Additive_type_3	100.0%	object
Additive3_percentage (wt%)	98.3%	float64
Sample_shape/Morphology	100.0%	object
PHA_degrading_microbes	100.0%	object
Experimental_Scale	100.0%	object

**Table 6:** Category Grouping of the feature “Degradation Environment”.

Original Category	Count	Merged Category	Count
Soil	572	Soil	586
field soil	14	Soil	
marine	508	Marine/Aquatic	608
freshwater	100	Marine/Aquatic	
industrial compost	126	Compost/Organic Fertilizer	250
compost	110	Compost/Organic Fertilizer	
vermicompost	4	Compost/Organic Fertilizer	
thermophilic compost	4	Compost/Organic Fertilizer	
organic fertilizer	6	Compost/Organic Fertilizer	
liquid mineral medium	102	Laboratory/Mineral Media	102

**Table 7:** Summary of Category Grouping for the categorical features.

Feature	No. of Initial Categories	No. of Merged Categories
Degradation_mechanism	9	4
Degradation_Environment	10	4
Additive_type_1	7	5
Sample_shape/Morphology	15	5
PHA_degrading_microbes	18	5

**Table 8:** Model performance without outlier exclusion.

Model	Set	R^2^	RMSE	MAE
Random Forest	Training	0.969	5.91	2.93
	Test	0.925	8.98	5.12
XGBoost	Training	0.967	6.07	3.52
	Test	0.922	9.16	5.32

**Table 9:** Optimized Hyperparameter Values for both regressors.

Random Forest		XGBoost	
Parameter	Best Value	Parameter	Best Value
max_depth	10	max_depth	6
min_samples_leaf	1	learning_rate	0.01
min_samples_split	5	colsample_bytree	0.8
n_estimators	200	n_estimators	800
		subsample	0.7

**Table 10:** Performance of tuned models without outlier exclusion.

Metric	Random Forest (Tuned)	XGBoost (Tuned)
Train R^2^	0.955	0.959
Test R^2^	0.930	0.931
CV Mean R^2^	0.887	0.884
CV SD R^2^	0.039	0.040

**Table 11:** Sensitivity analysis on the effect of the imputed degradation temperature (t_deg).

Model	t_teg	Train R^2^	Test R^2^
Random Forest (Tuned)	With	0.955	0.930
	Without	0.948	0.918
XGBoost (Tuned)	With	0.959	0.931
	Without	0.950	0.918

**Table 12:** Model performance on the unseen dataset.

Model	R^2^ (Unseen Dataset)
Random Forest	0.716
XGBoost	0.829

**Table 13:** Key features influencing PHBV-based formulations’ weight loss as derived from ML models.

Feature	Influence on Weight Loss
Degradation Environment	This environmental factor was identified as the most influential factor in the RF model. Specifically, the soil environment was associated with significantly reduced predicted weight loss compared to other environments. This was attributed to soil’s restrictive nature, which included limited oxygen diffusion and heterogeneous moisture distribution.
Degradation Time	This temporal factor was identified as a dominant feature, especially in the XGBoost model. Naturally, weight loss increased over time as the physical disintegration of the polymer progressed.
Adjusted H/B Ratio	Polymer composition, specifically the HB ratio, played a critical role. The models indicated that the specific chemical structure and ratio of monomers in the PHBV-based formulations were key predictors of how quickly the material physically lost mass.
Additive Presence and Type	The inclusion of additives, such as natural fibers (e.g., wood flour) or plasticizers, significantly modified the degradation rate. For instance, natural fibers enhanced degradation by increasing water uptake and microbial accessibility.

**Table 14:** Comparison of model performance with and without outlier exclusion.

Model	Outlier Exclusion	Train R^2^	Test R^2^	CV Mean R^2^	CV SD R^2^	Unseen R^2^
Random Forest	No	0.955	0.930	0.887	0.039	0.716
	Yes	0.958	0.911	0.890	0.015	0.741
XGBoost	No	0.959	0.931	0.884	0.040	0.829
	Yes	0.956	0.897	0.884	0.014	0.856

## Data Availability

The datasets used for training and evaluating the ML models were derived from curated biodegradation data reported in the literature and compiled within the framework of this study. The implementation of the ML models, including data preprocessing, feature selection, model training, and evaluation scripts, is publicly available via GitHub at: https://github.com/FSL-AUA/Weight-loss-model.git, accessed on 27 January 2026. In addition, the trained models and their associated feature sets are accessible through the Jaqpot platform: Weight or mass loss regression model A.N.I.P.H. (Jaqpot ID: 2343): https://app.jaqpot.org/dashboard/models/2343/description, accessed on 27 January 2026.

## Abbreviations

The following abbreviations were used in this manuscript:

ML	Machine Learning
QSAR	Quantitative Structure-Activity Relationship
FAMD	Factorial Analysis of Mixed Data
IQR	Interquartile Range
RMSE	Root Mean Square Error
MAE	Mean Absolute Error
XGBoost	Extreme Gradient Boosting
SHAP	Shapley Additive Explanations
ATBC	Acetyl Tributyl Citrate
CaCO_3_	Calcium Carbonate
CO_2_	Carbon Dioxide
CV	Cross-Validation
DDGS	Distillers Dried Grains with Solubles
ENR	Epoxidized Natural Rubber
HB	Hydroxybutyrate
HV	Hydroxyvalerate
ISO	International Organization for Standardization
kDa	Kilodalton
Mn	Number-Average Molecular Weight
Mw	Weight-Average Molecular Weight
Mv	Viscosity-Average Molecular Weight
PBAT	Poly(butylene adipate-co-terephthalate)
PCL	Poly(ε-caprolactone)
PDI	Polydispersity Index
PEO	Polyethylene Oxide
PHA	Polyhydroxyalkanoate
PHAs	Polyhydroxyalkanoates
PHBV	Poly(3-hydroxybutyrate-co-3-hydroxyvalerate)
PLA	Polylactic Acid
PRISMA	Preferred Reporting Items for Systematic Reviews and Meta-Analyses
R^2^	Coefficient of Determination
TEC	Triethyl Citrate
TOCA	Total Organic Carbon Availability
VS	Volatile Solids
WF	Wood Flour

## Appendix A

Table A1 reports the material features included in the PHBV biodegradation-induced weight/mass loss database, which is uploaded to the AUA Zenodo repository [38]. For each feature, the table provides the feature name as used in the dataset, a concise scientific description, the measurement unit when applicable, and the observed value range across all samples. The materials properties dataset comprises 46 distinct features (23 categorical & 23 numerical) describing material descriptors encompassing composition, additives, molecular descriptors, physicochemical properties, morphological characteristics, and surface characteristics of PHBV-based materials. The data were obtained from references [6,7,22,23,24,25,26,27,28,29,30,31,32,33,34,35,36].

**Table A1:** Overview of materials descriptors (independent variables) of the PHBV-based biodegradation-induced weight/mass loss database.

Feature Name	Description	Unit	Range	Type
Study_id	Unique identifier for study instance	-	varies	categorical
Instance	Experimental run number	-	varies	categorical
Sample_name	Identifier of the PHBV-based sample or formulation	–	varies	Categorical
Sample ratio	Weight-to-weight ratio of components in the formulation (wt/wt)	ratio, %	0–100	Categorical
Monomer_A	Primary monomer composing the PHBV copolymer	–	varies	Categorical
Monomer_B	Secondary monomer composing the PHBV copolymer	–	varies	Categorical
Adjusted_HB_ratio_formulation	HB ratio in formulation	mol%	14–99	Numerical
Adjusted_HV_ratio_formulation	Hydroxyvalerate (HV) ratio in formulation	mol%	0.5–19	Numerical
Additives	Presence of additives in the formulation	–	Yes/No	Categorical
Additive1_name	Name of the first additive	–	varies	Categorical
Additive_type_1	Type of the first additive	–	varies	Categorical
Additive1_percentage	Weight fraction of the first additive	wt%	0–100	Numerical
Additive2_name	Name of the second additive	–	varies	Categorical
Additive_type_2	Type of the second additive	–	varies	Categorical
Additive2_percentage	Weight fraction of the second additive	wt%	0–100	Numerical
Additive3_name	Name of the third additive	–	varies	Categorical
Additive_type_3	Type of the third additive	–	varies	Categorical
Additive3_percentage	Weight fraction of the third additive	wt%	0–100	Numerical
PHBV_weight_percentage_final_formulation	PHBV content in the final formulation	wt%	0–100	Numerical
Viscocimetric_molar_mass, Mv	Molar mass by viscosity	g/mol	128–155	numerical
Soil_burial_time_for_Mv	Soil burial time before test	months	0–3	numerical
Mw	Weight-average molecular weight	kDa	375–690	Numerical
Soil_burial_time_for_Mw (months)	Soil burial time before test	months	0–12	numerical
Relative Mw (%)	Relative molecular weight	%	29–100	numerical
Incubation_time_for_relative_Mw (days)	Incubation period for relative Mw	days	1–27	numerical
Mn	Number-average molar mass	kDa	111–350	numerical
Soil_burial_time_for_Mn (months)	Soil burial time before test	months	0–12	numerical
PDI	Polydispersity index	ratio	2.2–2.9	numerical
Soil_burial_time_for_PDI (months)	Soil burial time before test	months	0–12	numerical
Moisture_content_formulations (%)	Moisture in the formulation	%	0–19	numerical
Soil_burial_time _for_moisture_content (months)	Soil burial time before test	months	0–12	numerical
Density	Density of PHBV-based material	g/cm^3^	0.79–1.25	Numerical
Void_content (%)	Void content in the sample	%	0–5	numerical
Sample_shape/Morphology	Physical shape or morphology of the PHBV-based sample (e.g., film, pellet, fiber, composite)	-	varies	Categorical
Size_diameter	Diameter of the PHBV sample or morphological element (e.g., particle, sphere, or cylindrical structure), as reported in the source studies	cm	9.0	Numerical
Size_length	Length of the PHBV sample or morphological element (e.g., films or tubes), as reported in the source studies	cm	2.5–20	Numerical
Size_Width	Width of the PHBV sample or morphological element (e.g., films), as reported in the source studies	cm	1–20	Numerical
Size_Thickness	Thickness of the PHBV sample or morphological element (e.g., films), as reported in the source studies	cm	0.01–0.3	Numerical
Water_absorption_capacity	Water absorption capacity	%	0.7–33	Numerical
Immersion_days	Duration of exposure	days	1–9	Numerical
Thickness_swelling (%)	Thickness swelling after immersion	%	0.96–6.5	numerical
Immersion_days	Duration of exposure	days	1–9	Numerical
Film_solubility	Solubility of PHBV films	%	1–21	Numerical
Static_water_contact_angle	Static water contact angle indicating hydrophilicity	deg	57–83	Numerical

Table A2 summarizes the environmental and experimental conditions under which PHBV biodegradation experiments were conducted, which were included in the biodegradation database uploaded to the AUA Zenodo repository [38]. For each feature, the table reports the feature name, a concise description, measurement unit (when applicable), the observed range across all studies and the feature type (categorical or numerical). Environmental conditions were described using 29 features, including 13 numerical and 16 categorical variables encompassing biochemical condition, the physical medium, temperature, pH, moisture, salinity, nutrient content, solids composition, microbial presence, and standardized testing protocols. The data were obtained from references [6,7,22,23,24,25,26,27,28,29,30,31,32,33,34,35,36].

**Table A2:** Overview of environmental and experimental features (independent variables) of the PHBV-based biodegradation-induced weight/mass loss database.

Feature Name	Description	Unit	Range	Type
Sample_name	Identifier of the PHBV-based sample tested	–	varies	Categorical
Parameter_evaluated	Environmental parameters evaluated in the study	–	varies	Categorical
Degradation_condition	General biodegradation condition (e.g., aerobic, anaerobic) soil, marine, compost)	–	varies	Categorical
Degradation_mechanism	Dominant biodegradation mechanism reported	–	varies	Categorical
T_biodeg	Temperature during the biodegradation experiment	°C	20–60	Numerical
T_biodeg_units	Unit used to report biodegradation temperature	–	celsius	Categorical
T_biodeg_winter_marine	Marine biodegradation temperature under winter conditions	°C	8–15	Numerical
T_biodeg_summer_marine	Marine biodegradation temperature under summer conditions	°C	20–25	Numerical
ΤH20_winter_marine	Water exposure time (T_h20_) in winter marine conditions	–	12–29	Numerical
ΤH20_summer_marine	Water exposure time (T_h20_) in summer marine conditions	–	27	Numerical
Water_pH_marine	pH of marine water during biodegradation	–	7.2–8.2	Numerical
Salinity_marine	Salinity of the marine environment	ppt	37–38	Numerical
Soil_moisture	Moisture content of soil	%	20–50.5	Numerical
Soil_pH	pH of soil during biodegradation	–	6.6–7.2	Numerical
Soil_T	Soil temperature during biodegradation	°C	22.7–29	Numerical
Compost_moisture	Moisture content of compost	%	55–81	Numerical
Compost_pH	pH of the compost environment	–	6–8.2	Numerical
Compost_T	Compost temperature during biodegradation	°C	21–58	Numerical
TOCA	Total organic carbon availability	%	0.13–0.55	Numerical
C_N_ratio	Carbon-to-nitrogen ratio of the environment	–	18–28.9	Numerical
VS	Volatile solids fraction of the biodegradation environment	%	26.6	Numerical
PHA_degrading_microbes	Presence or abundance of PHA-degrading microorganisms	–	varies	Categorical
Degradation_Environment	Classified degradation environment	–	varies	Categorical
ASTM/ISO	Standard used for biodegradation testing	–	varies	Categorical

Table A3 summarizes the biodegradation response variables included in the PHBV biodegradation database uploaded to the AUA Zenodo repository [38]. For each feature, the table reports the feature name, a concise description, the measurement unit, the observed value range across all studies, and whether the feature is categorical or numerical. The data were obtained from references [6,7,22,23,24,25,26,27,28,29,30,31,32,33,34,35,36].

**Table A3:** Overview of weight loss features of the PHBV-based biodegradation database.

Feature Name	Description	Unit	Range	Type
Sample_name	Identifier of the PHBV-based sample	–	ranges	Categorical
Weight_loss_mass_loss_percentage (Dependent Target value)	Lost mass due to biodegradation	%	0–100	Numerical

Table A4 summarizes the temporal variables used to describe the progression of PHBV biodegradation included in the biodegradation dataset uploaded to the AUA Zenodo repository [38]. For each feature, the table reports the feature name, a concise description, the measurement unit, the observed value range across all studies, and whether the feature is categorical or numerical. The data were obtained from references [6,7,22,23,24,25,26,27,28,29,30,31,32,33,34,35,36].

**Table A4:** Overview of time-point features of the PHBV-based biodegradation database.

Feature Name	Description	Unit	Range	Type
Sample_name	Identifier of the PHBV-based sample	–	–	Categorical
Biodegradation_time	Time at which the weight or mass loss was measured	Days	0–360	Numerical

The numerical input ranges reported in Table A5 corresponded to the minimum and maximum values observed in the final training dataset used for model development. These ranges defined also the recommended limits for user inputs in the Jaqpot platform to avoid predictions outside the model’s applicability domain (the reported ranges are also visible in the platform).

**Table A5:** Numerical input ranges used for the implementation of the QSAR model.

Feature Name	Unit	Range
Additive1_percentage	wt%	0–70
Additive2_percentage	wt%	0–50
Additive3_percentage	wt%	0–20
Adjusted_HB_ratio_formulation	mol%	28–99
Adjusted_HV_ratio_formulation	mol%	0.5–19
T_deg	°C	20–60
Degradation_time	days	0–360
